# Coaxial liver biopsy with tract electrocauterization: technique and outcomes in high-risk patients with suspected hepatocellular carcinoma

**DOI:** 10.1007/s00261-026-05496-z

**Published:** 2026-04-03

**Authors:** Tolga Zeydanli, Muhammet Kursat Simsek, Ozgur Ozen, Batuhan Kirisci, Adem Safak, Fatih Boyvat, Mehmet Haberal

**Affiliations:** 1 Department of Radiology, Başkent University, Ankara, Turkey; 2 Department of General Surgery, Başkent University, Ankara, Turkey

**Keywords:** Hepatocellular carcinoma, Liver biopsy, Coaxial technique, Electrocauterization, Tract seeding, Bleeding complications, High-risk patients

## Abstract

**Purpose:**

To evaluate the safety and efficacy of a technique combining coaxial biopsy with electrocauterization of the biopsy tract in high-risk patients with suspected hepatocellular carcinoma (HCC).

**Methods:**

We retrospectively analyzed 102 liver biopsies performed in 99 patients (78 males, 21 females) with suspected HCC using a 17-gauge coaxial introducer needle and 18G automatic biopsy needle. After sample collection, the biopsy tract was sealed by applying monopolar electrocauterization to the coaxial needle during its removal. Patient demographics, procedural details, contraindications (INR > 1.5, platelet count < 50,000/mm³, ascites, antiplatelet/anticoagulant use), pathology results, and complications were recorded. Follow-up imaging (range: 18–360 days) was systematically reviewed to identify potential tumor seeding along the biopsy tract. All procedures were performed under ultrasound guidance with 4-h post-procedure observation.

**Results:**

All 102 biopsies yielded 100% diagnostic success rate with cirrhosis in 85.2% and HCC in 73.5% of patients. Most patients (75.8%) had at least one contraindication for standard biopsy. We observed one mortality in a high-risk Jehovah's Witness patient and one minor complication (hemoglobin drop) managed conservatively. Among 52 patients with follow-up imaging (mean 149 days), one case of suspected intrahepatic tract seeding occurred with no extrahepatic seeding.

**Conclusion:**

This refined electrocauterization technique provides a safe, effective approach for obtaining diagnostic tissue in high-risk HCC patients with contraindications to standard percutaneous biopsy, offering advantages over conventional tract embolization methods through precise thermal effect localization.

## Introduction

Hepatocellular carcinoma (HCC) ranks as the sixth most prevalent cancer globally and stands as the third leading cause of cancer-related mortality, as well as the primary cause of death in patients with cirrhosis [1]. These numbers are expected to rise in the coming decades, with projections estimating that more than 1 million people will die due to liver cancer in 2030 [2].

According to current guidelines, liver biopsy plays a limited role in HCC management. In patients with liver cirrhosis, a non-invasive diagnosis is highly specific if typical imaging features are present. However, biopsy is recommended for patients without cirrhosis or when lesions in cirrhotic patients lack characteristic radiologic features [3]. In most cases, liver biopsy, which carries a risk of bleeding and tumor seeding, is not required for routine HCC diagnosis. However, the discussion on broader biopsy application remains active [4], as molecular and immunohistochemical insights have gained increasing importance in the era of personalized oncology. Recent therapeutic advancements necessitate molecular markers for tailoring systemic treatments [5], particularly for advanced-stage HCC [6]. The lack of tissue samples in a significant proportion of HCC cases poses a major challenge, as effective personalized medicine relies on integrative molecular and morphological tumor subtyping [7].

Core biopsy can be valuable for evaluating the molecular profile to guide potential targeted therapies or for storing tissue samples for emerging therapeutic developments [4, 8]. However, patients with suspected HCC are at increased risk for biopsy-related complications due to multiple factors. Most patients are cirrhotic, which predisposes them to thrombocytopenia, elevated INR, and ascites. Additionally, the hypervascular nature of most HCC lesions further increases the risk of hemorrhagic complications [9, 10]. Additionally, HCC carries an inherent risk of tumor seeding along the biopsy tract [11].

Given the increasing demand for core biopsies in this high-risk patient population, mitigating these risks is crucial. To address these challenges, we adopted and further refined a technique combining the coaxial biopsy system with electrocauterization of the biopsy tract through the coaxial needle. Coaxial biopsy is considered safer compared to other techniques for reducing needle seeding risk [12, 13], as it allows for the collection of multiple tissue samples from the lesion using a single puncture [14]. Electrocauterization has long been used for hemostasis across various surgical fields and it has also been suggested for tract sealing in very few animal and human studies [15–17].

We hypothesized that applying electrocauterization through the coaxial needle after biopsy could control bleeding and reduce the risk of tumor seeding by sealing the biopsy tract. In this study we present the seven-year results of this method in high-risk patients with suspected HCC lesions.

## Materials and methods

### Technique

**Fig. 1:**
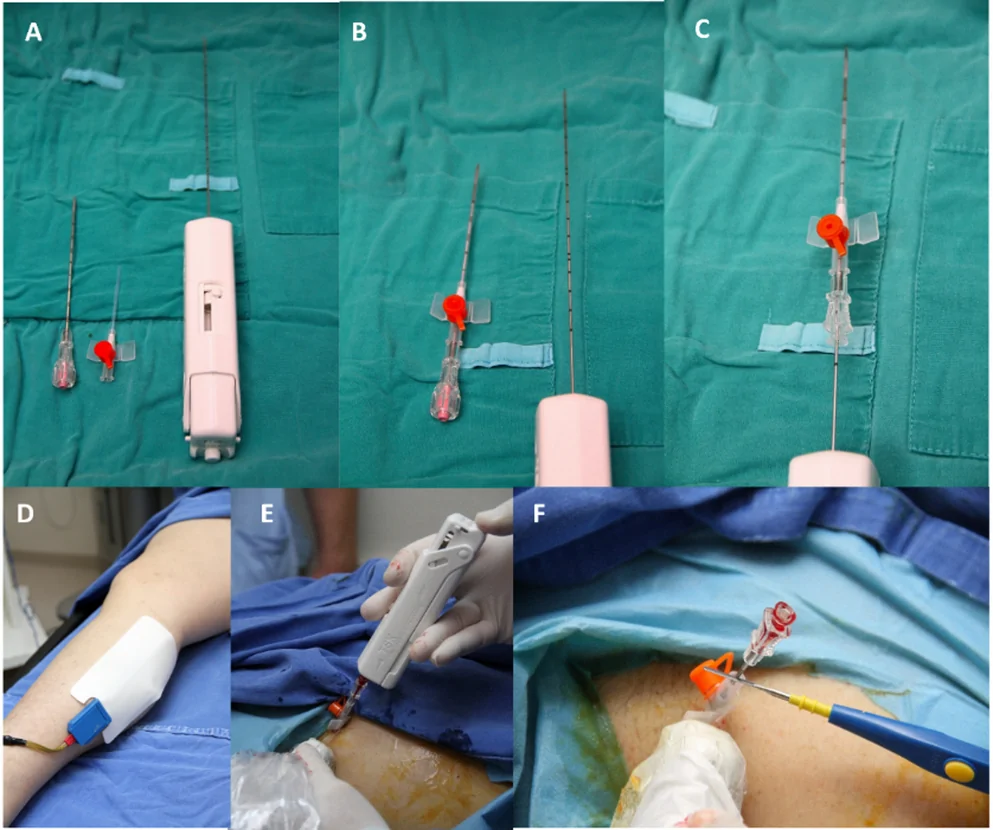
Procedural setup and technique for electrocautery assisted coaxial liver biopsy. **A** Required materials: 17-gauge coaxial needle, intravenous cannula, and 18-gauge automatic biopsy needle. **B** Coaxial needle inserted through the intravenous cannula prior to skin puncture to insulate skin from cauterization effects. **C** 18G biopsy needle advanced through coaxial needle sheathed with I.V. cannula **D** Grounding pad attached to the patient’s leg. **E** Multiple tissue samples obtained through the coaxial needle after coaxial needle positioned at the target site. **F** Electrocautery was applied to the needle shaft between the hub and intravenous cannula during needle withdrawal to seal the biopsy tract

**Fig. 2:**
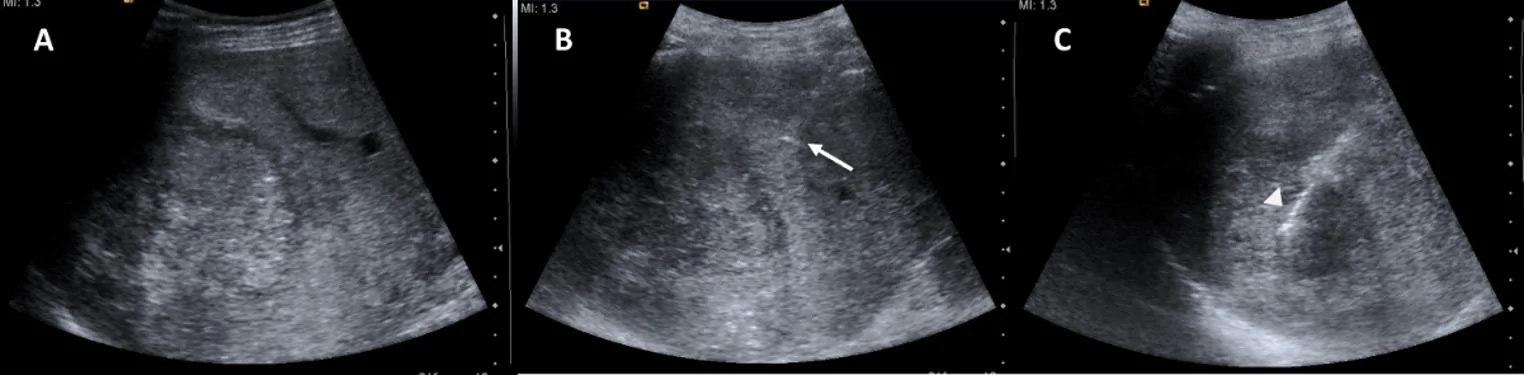
Ultrasound-guided biopsy procedure in a patient with hepatocellular carcinoma. **A** Ultrasound image demonstrating a right lobe hepatic mass. **B** Ultrasound image showing the coaxial needle positioned at the peripheral margin of the target lesion (arrow). **C** Ultrasound image documenting tissue sampling with an 18-gauge biopsy needle advanced through the coaxial system (arrowhead)

All procedures were performed under sterile conditions with sedation and analgesia (propofol, fentanyl, and midazolam) and local anesthesia (lidocaine). A 17-gauge, 9.9 cm coaxial introducer needle (Argon Medical Devices, TX, USA) was insulated using a 14-gauge I.V. cannula to protect the skin from cauterization effects (Fig. 1A). Under ultrasound guidance (Acuson S2000, Siemens Diagnostic Ultrasound System, USA; probes 6C1 and 9L4), the coaxial needle was advanced into the liver. If liver parenchyma biopsy was required, it was performed first, followed by directing the 17-gauge needle to the target liver mass without exiting the liver capsule. During the biopsy, the needle tract was continuously monitored using ultrasound to ensure accurate sampling of the target lesion while avoiding injury to major vessels (Fig. 2). Tissue samples were obtained using an 18-gauge, 15-cm automatic biopsy needle (Acecut, TSK Laboratory, Japan) through the 17G guiding needle. An adequate number of samples were obtained at the operator’s discretion to ensure a definitive pathological diagnosis.

After completion of sample collection, electrocautery at 40 W was applied to the coaxial needle shaft between the needle hub and the intravenous cannula (Fig. 1F) during needle withdrawal to achieve hemostasis and reduce the risk of tumor seeding. The coaxial needle was withdrawn gradually under continuous ultrasound guidance at a rate of 1 s per centimeter of tract, with electrocautery applied continuously throughout the withdrawal. Electrocautery was discontinued once the needle tip exited the liver capsule to avoid thermal injury to the skin and subcutaneous tissues. All procedures were carried out by two interventional radiologists with at least ten years of experience in a single institution. Patients were observed for at least 4 h after the procedure and control hemoglobin tests were used to check for hemorrhagic complications.

For this technique, we employed monopolar electrocauterization with a single electrode applied to the needle and current passing through the body to a grounding pad attached to patient`s leg. Coagulation electrocautery mode at 40 watts was selected because its pulsed, lower-frequency current (with higher voltage) produces sufficient heat for cell desiccation and clot formation while minimizing tissue charring [15, 18]. This approach generates greater thermal spread into surrounding tissue, effectively sealing both the biopsy tract and any bleeding vessels [18–20].

**Fig. 3:**
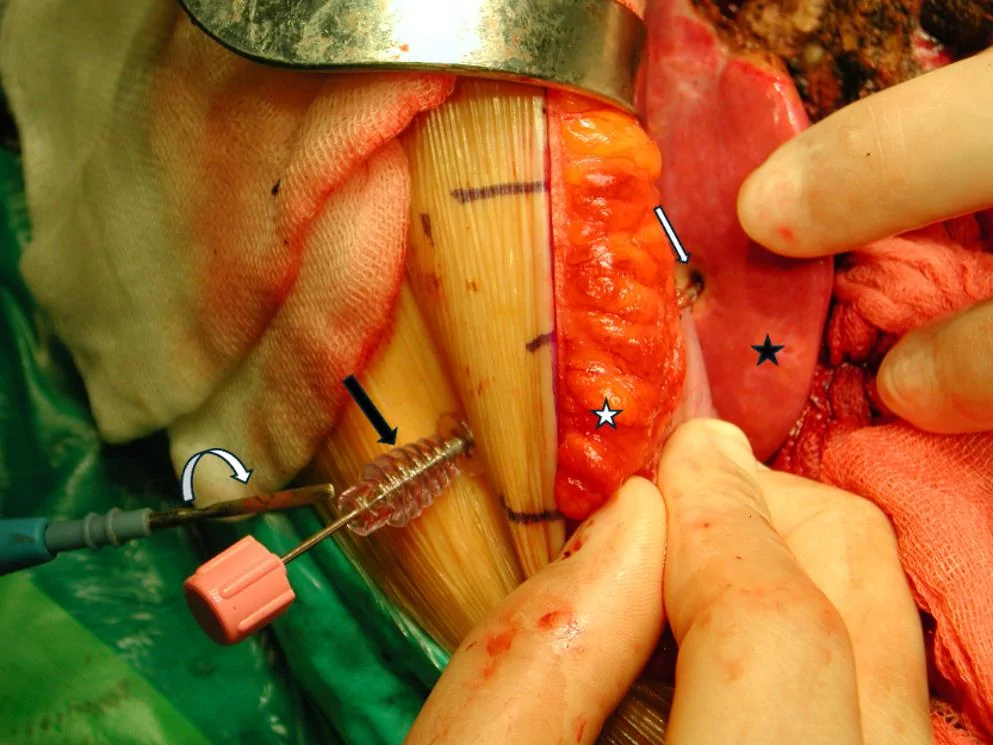
Intraoperative image of percutaneously placed needle during electrocautery application, demonstrating the effectiveness of tract sealing: Electrocautery probe (curved arrow), coaxial needle (black arrow), subcutaneous tissue (white star), visible cautery effect at the liver tissue (white arrow), the liver segment designated for resection (black star)

To evaluate the effect of electrocauterization through a coaxial needle, we initially tested the technique intraoperatively in five patients scheduled for liver lobectomy. The coaxial needle was inserted percutaneously into the liver tissue designated for resection. Electrocautery was applied during needle withdrawal, demonstrating the technique to be effective and free of adverse effects (Fig. 3). Based on these findings, we incorporated electrocauterization into our coaxial biopsy procedures in high-risk patients.

### Patient selection and data analysis

This single-institution, retrospective study was approved by the university institutional review board and was conducted under the Declaration of Helsinki. Written informed consent was obtained from all patients prior to biopsy; for underage patients, consent was obtained from a parent or legal guardian. Following the initial intraoperative experience, biopsy with monopolar tract cauterization was adopted into our clinical practice in May 2016. The intraoperative validation cases were not included in the study cohort; all patients who participated in the intraoperative phase also provided written informed consent prior to the procedure. The hospital database was retrospectively reviewed to identify patients with suspected HCC lesions in diagnostic imaging who underwent liver biopsy using the coaxial technique with tract cauterization between May 2016 and September 2024.

Tract cauterization was used in patients considered to be at high risk of bleeding. High bleeding risk was defined by the presence of at least one of the following: hypervascular mass, cirrhotic liver, ascites, an international normalized ratio (INR) > 1.5, platelet count < 50,000/mm³, or ongoing antiplatelet or anticoagulant therapy. Coagulation abnormalities and antiplatelet or anticoagulant use were corrected or discontinued whenever feasible. If adequate correction could not be achieved, biopsy was nevertheless performed using the tract cauterization technique.

Patient demographics, INR, platelet counts, number of biopsy samples obtained, size of liver masses, pathology reports, presence of ascites, and use of anti-platelet and anti-coagulant medications were recorded. All patients were observed for at least 4 h post-procedure to monitor for complications, which were documented according to the Society of Interventional Radiology guidelines [21]. We systematically reviewed follow-up imaging and surgical reports to identify tumor seeding, defined as tumor deposits along the biopsy tract. For suspected seeding cases, two radiologists independently reviewed the images and reached consensus on the diagnosis.

The Statistical Package for Social Sciences (SPSS) version 26.0 for Windows (SPSS Inc., Chicago, IL, USA) was used for statistical analyses. Mean with standard deviation (SD), median with minimum and maximum values, and percentages were used for descriptive statistics. The distribution of variables was checked with the Kolmogorov-Smirnov test.

## Results

Between May 2016 and September 2024, 102 coaxial biopsies with tract cauterization were performed in 99 patients (78 men and 21 women) with suspected hepatocellular carcinoma. Three patients underwent multiple biopsies for newly developed lesions. Patient age ranged from 10 to 81 years (mean 61.8 years, SD 13.1). Detailed demographic and clinical characteristics are presented in Table 1.

**Table 1:** Demographics and clinical characteristics

Number of biopsies/patients	102/99
Gender	
M	79% (78/99)
F	21% (21/99)
Age (mean ± SD, range)	61.8 ± 13.1 (10–81)
Number of samples (median, range)	5 (2–14)
Cirrhosis	85.2% (87/102)
Mass diameter (mean ± SD, range)	65.38 ± 43.25 mm (10–215)
Diameter range (number of patients)	0–2 cm(*n* = 6,5.9%) 2–5 cm (*n* = 41,40.2%) 5–10 cm(*n* = 36,35.3%) >10 cm (*n* = 19,18.6%)

**Table 2:** Contraindications for liver biopsy in the study population

	Total (*n*)
High INR (> 1.5)	14
Low thrombocyte < 50,000/mm³	6
Ascites	65
Anti-platelet, coagulant use	26
Patients with ≥ 2 contraindications*	30

*Of 30 patients with multiple contraindications, 25 had two, 4 had three, and 1 had four concurrent contraindications

All biopsy specimens obtained during the procedures yielded adequate tissue for histopathological examination, resulting in a 100% diagnostic success rate. For each patient, one sample was obtained from the liver parenchyma and multiple samples from the target mass through the coaxial needle to optimize diagnostic accuracy. In four patients, the anatomical location of the lesion precluded a transhepatic approach, and direct puncture of the mass was performed instead. Among these four cases, one patient experienced the fatal hemorrhagic complication described below. The median number of core samples obtained was 5 (range: 2–14). Histopathological analysis confirmed cirrhosis in 87 patients (85.2%). Final pathological diagnoses were distributed as follows: hepatocellular carcinoma (*n* = 75, 73.5%), cholangiocarcinoma (*n* = 8, 7.8%), regenerative nodule (*n* = 5, 4.9%), metastatic disease (*n* = 4, 3.9%), neuroendocrine tumors (*n* = 3, 2.9%), mixed hepatocellular-cholangiocarcinoma (*n* = 2, 2.0%), lymphoma (*n* = 2, 2.0%), chronic inflammation (*n* = 2, 2.0%), and amyloidosis (*n* = 1, 1.0%).

One patient in this cohort was a 10-year-old child with a known history of glycogen storage disease who presented with a hepatic lesion lacking definitive imaging characteristics for a specific diagnosis. Given the absence of pathognomonic imaging features, biopsy was indicated to establish a histopathological diagnosis. Final pathology confirmed hepatocellular carcinoma, consistent with the well-documented association between glycogen storage disease and HCC development even in the pediatric population.

Of the 99 patients, 75 (75.8%) presented with at least one relative contraindication for liver biopsy: INR > 1.5, platelet count < 50,000/mm³, ascites, or concurrent antiplatelet/anticoagulant use (Table 2). Thirty patients (30.3%) had two or more concurrent contraindications.

We observed one major complication resulting in mortality. A 68-year-old male patient with cirrhosis, ascites, coagulopathy (INR 2.06), and severe thrombocytopenia (platelets 32,000/mm³) — who declined pre-procedural transfusion support on religious grounds as a Jehovah’s Witness — underwent biopsy of a 75 × 67 mm hypervascular lesion (final pathology HCC) located at the lateral aspect of segment 7. The exophytic location of the mass precluded a transhepatic approach, as there was no feasible tract traversing normal liver parenchyma prior to entering the lesion. Transjugular liver biopsy was considered but deemed technically unfeasible given the peripheral exophytic location of the mass. Coaxial biopsy with tract cauterization was therefore performed via direct puncture of the mass following multidisciplinary consensus and with documented patient consent. Following the procedure, hemorrhagic complications were identified during post-procedural monitoring. Despite emergency surgical intervention, during which active bleeding at the biopsy site on the mass capsule was identified and controlled with packing, the patient succumbed to hemorrhagic shock on the first postoperative day, as blood product transfusion could not be administered in accordance with the patient’s documented wishes. We documented one minor complication consisting of right upper quadrant pain and a hemoglobin decrease of 2 g/dL, which resolved with supportive care. No additional complications were observed in the remaining patients. Due to the low overall complication rate observed in this series — with only one major complication event recorded among 102 procedures — formal subgroup analyses and regression modeling of complication predictors were not statistically feasible.

Follow-up imaging to assess for tumor seeding was available for 52 (52.5%) of the 99 patients. The follow-up period ranged from 18 to 360 days (mean: 149 days, SD: 71). Only one patient demonstrated radiological findings suggestive of tumor seeding. This patient initially presented with a single 69 × 55 mm lesion in segment 6 of the liver at the time of biopsy, later underwent selective trans-arterial chemoembolization. At the 6-month follow-up imaging, disease progression was evident with multiple new lesions demonstrating imaging features compatible with HCC in both hepatic lobes. Additionally, a 5 cm subcapsular mass was identified adjacent to the necrotic index lesion, along the presumed biopsy tract, suspicious for tract seeding. There was no extrahepatic involvement.

## Discussion

Liver biopsy continues to play a critical role in the diagnosis and prognostication of HCC, despite procedure-associated complications and limitations. While advancements in imaging technologies have restricted biopsy applications to specific clinical scenarios, recent investigations into tissue biomarkers, molecular classifications, and targeted therapies have demonstrated that morphologic variants and subtypes can function as reliable surrogates for molecular signatures. This evidence has reinvigorated the scientific community’s focus on tissue analysis, elevating tumor biopsy as an essential resource for HCC diagnosis, management, and prognostication [4–8, 22]. However, the procedural safety of liver biopsy in high-risk patients with coagulopathies or hypervascular lesions remains a significant clinical concern.

Historically, percutaneous HCC biopsies were associated with relatively low diagnostic yield (86–91%), high rate of serious bleeding complications (1.1%), and significant risk of extrahepatic tumor seeding (1.6–2.6%) even in patients without any contraindications to percutaneous biopsy [23, 24]. The introduction of the coaxial technique has since improved safety profiles by allowing multiple samples to be collected through a single parenchymal puncture [12, 25, 26]. Schaffler-Schaden et al. [25] reported only one serious bleeding complication among 131 patients with normal coagulation parameters undergoing coaxial biopsy for focal liver lesions, with no tumor seeding. Similarly, Maturen et al. [12] reported a 3.9% serious bleeding complication rate with no seeding using the coaxial technique exclusively in HCC patients with normal bleeding parameters. It is worth noting that both series were conducted in patients without significant bleeding risk factors, whereas 75.8% of patients in our cohort presented with at least one contraindication. Despite this substantially higher-risk profile, we observed a major complication rate of only 0.98% (1/102), which compares favorably with complication rates reported in lower-risk coaxial biopsy series. Various embolization methods have been developed to overcome biopsy-related complications in high-risk patients. These include plugging the needle tract with gelfoam [27, 28], N-butyl cyanoacrylate (NBCA) [29], or coils [30]. Tract embolization with gelfoam was first described in 1984 by Riley et al. [27], remains the most widely documented approach, with studies reporting overall success rate around 95% and complication rates below 2% [31–33]. Singhal et al. [28] reported no serious complication in a large series of 782 patients. NBCA embolization has also demonstrated favorable outcomes, with one study reporting no serious complications among 86 patients (including 21 with relative contraindications) [29], Kim et al. [34] similarly reported no serious complications with NBCA. In a study in which coils were used as an embolic agent, Rathod et al. [35] reported 3 serious bleeding complications out of 127 biopsies. While direct comparisons are limited by differences in patient populations and study designs, our major complication rate of 0.98% using electrocauterization, is comparable to complication rates reported with Gelfoam and coil embolization in lower-risk cohorts. Furthermore, liquid embolic agents like NBCA carry the risk of non-targeted embolization into portal or hepatic veins and require significant technical expertise [36]. Electrocauterization offers a potential advantage by limiting the sealing effect precisely to the needle tract, thereby potentially reducing off-target embolization while requiring a less steep learning curve compared to liquid embolic techniques.

While early animal models first suggested the utility of electrocauterization for tract sealing, human clinical data remain limited [15, 16]. Shyn et al. [37] reported no major complications in a 41-patient cohort using a dedicated bipolar radiofrequency device, and confirmed the success of bipolar radiofrequency device across various organ systems in another study [38]. Similarly, Song et al. [39] demonstrated effective tract sealing using monopolar radiofrequency device in 10 patients with normal coagulation profiles.

Our study significantly expands this evidence base, representing the largest clinical series to date with 102 procedures. Unlike previous studies that utilized specialized, proprietary RF hardware, our technique relies on standard surgical cautery. Because this equipment is readily available in medical centers, the approach can be seamlessly integrated into existing coaxial biopsy protocols without additional capital investment or specialized training.

The clinical impact of this technique is underscored by our high-risk cohort: 85.2% of our patients were cirrhotic, and nearly one-third presented with multiple concurrent bleeding risk factors. Such patients are traditionally excluded from percutaneous biopsy unless complex embolization methods are employed. Despite this high-risk profile, we observed only one serious bleeding complication (0.98%). The single mortality case in our series warrants careful analysis, as it carries direct implications for patient selection criteria and procedural planning. Regarding this case, the decision to proceed with biopsy was made following multidisciplinary consensus, as histopathological confirmation was considered essential for treatment planning; the imaging findings were not definitively diagnostic, and tissue acquisition was required to determine the patient’s eligibility for locoregional therapy.

From a technical standpoint, the exophytic location of the mass necessitated direct puncture without a traversing parenchymal tract, which may have compromised the effectiveness of tract cauterization in two ways: first, the absence of a parenchymal sealing surface reduced the mechanical effectiveness of thermal coagulation; and second, the heterogeneous, partially necrotic composition of the mass may have further impaired adequate thermal sealing. Notably, direct mass puncture was required in three additional patients — one with ascites and normal coagulation parameters (final pathology: metastasis) and two on ongoing clopidogrel therapy with otherwise normal coagulation parameters (final pathology: HCC in both) — none of whom experienced hemorrhagic complications. This suggests that direct mass puncture per se does not inevitably increase bleeding risk, and that the fatal outcome in the index case was likely attributable to the compounding effect of multiple concurrent high-risk features rather than the direct puncture technique alone. Nevertheless, this case highlights an important limitation that tract cauterization may be less effective when a transhepatic parenchymal tract cannot be established, and extreme caution is warranted when direct mass puncture is required in the setting of severely deranged coagulation parameters.

Our results suggest that coaxial biopsy with tract cauterization may represent a promising strategy for HCC evaluation in selected high-risk patients. With further prospective validation in adequately powered comparative studies, this technique may potentially contribute to a reassessment of current risk thresholds for percutaneous biopsy. In carefully selected patients, diagnostic biopsies may be feasible without the standard requirement of suspending anticoagulants or administering prophylactic blood products.

Tumor seeding is defined as the presence of neoplastic cells along the needle tract, typically manifesting as nodular tissue outside the liver capsule in the peritoneal cavity, subcutaneous tissue, abdominal muscles, or skin. The interval between biopsy and seeding detection varies considerably, reported as early as 21 days post-procedure [40], but with a median detection time of 13 months [41]. In our cohort, post-biopsy intrahepatic seeding was suspected in one patient along the biopsy tract in the setting of advanced, aggressive disease, while no extrahepatic seeding was identified in available follow-up imaging or surgical reports. However, these findings must be interpreted with considerable caution: follow-up imaging was available for only 52.5% of patients, and the mean follow-up of 149 days falls substantially short of the reported median seeding detection interval of 13 months, meaning that delayed seeding events may not yet have been clinically apparent. The typically limited survival of advanced HCC patients and the confounding effects of subsequent locoregional treatments further complicate interpretation. Prospective studies incorporating standardized long-term imaging follow-up will be necessary to adequately assess the seeding risk associated with this technique.

Our study has several limitations. First, its retrospective, single-arm design and the absence of a comparator cohort limit causal inference and preclude direct comparison of complication rates with alternative tract-sealing techniques. Randomized controlled trials comparing this technique with standard percutaneous biopsy or alternative embolization methods would provide more robust evidence. Second, the very low complication rate rendered formal subgroup analyses and regression modeling of complication predictors statistically infeasible; prospective, adequately powered studies will be necessary to identify predictors of procedural risk. Third, the absence of a standardized follow-up protocol for seeding assessment and the relatively short mean follow-up period constrain our ability to definitively exclude delayed tract seeding events.

## Conclusion

In conclusion, the findings of this study suggest that coaxial biopsy with tract cauterization may represent a technically feasible and potentially effective approach for obtaining diagnostic tissue samples in high-risk HCC patients with coagulopathies. The technique demonstrated excellent diagnostic yield with an acceptable complication profile in this challenging patient population; however, prospective comparative studies with standardized follow-up protocols will be necessary to more definitively establish its safety and efficacy.

## Data Availability

No datasets were generated or analysed during the current study.
